# Reactive Oxygen Species and Antitumor Immunity—From Surveillance to Evasion

**DOI:** 10.3390/cancers12071748

**Published:** 2020-07-01

**Authors:** Andromachi Kotsafti, Marco Scarpa, Ignazio Castagliuolo, Melania Scarpa

**Affiliations:** 1 Laboratory of Advanced Translational Research, Veneto Institute of Oncology IOV-IRCCS, 35128 Padua, Italy; andromachi.kotsafti@iov.veneto.it; 2 General Surgery Unit, Azienda Ospedaliera di Padova, 35128 Padua, Italy; marcoscarpa73@yahoo.it; 3 Department of Molecular Medicine DMM, University of Padua, 35121 Padua, Italy; ignazio.castagliuolo@unipd.it

**Keywords:** reactive oxygen species, oxidative stress, immunity, inflammation, cancer

## Abstract

The immune system is a crucial regulator of tumor biology with the capacity to support or inhibit cancer development, growth, invasion and metastasis. Emerging evidence show that reactive oxygen species (ROS) are not only mediators of oxidative stress but also players of immune regulation in tumor development. This review intends to discuss the mechanism by which ROS can affect the anti-tumor immune response, with particular emphasis on their role on cancer antigenicity, immunogenicity and shaping of the tumor immune microenvironment. Given the complex role that ROS play in the dynamics of cancer-immune cell interaction, further investigation is needed for the development of effective strategies combining ROS manipulation and immunotherapies for cancer treatment.

## 1. Introduction

Reactive oxygen species (ROS) are defined as chemically reactive derivatives of oxygen that elicits both harmful and beneficial effects in cells depending on their concentration. Oxidative stress occurs when ROS production overcomes the scavenging potential of cells or when the antioxidant response is severely impaired; as a consequence nonradical and free radical ROS such as hydrogen peroxide (H_2_O_2_), the superoxide radical (O_2_^●^) or the hydroxyl radical (OH^●^) accumulate [1]. They can represent by-products of mitochondrial adenosine triphosphate generation in the electron transport chain or they can be produced in enzymatic reactions mainly mediated by the NADPH oxidase (NOX) and Dual Oxidase (DUOX) families, while the antioxidative machinery include enzymes such as superoxide dismutase (SOD), catalase (CAT) and glutathione peroxidase (GPX) [2]. 

Although oxidative stress can cause toxicity, it is essential to realize that redox signaling is pivotal for critical functions in physiological systems and immunity against disease. Indeed, ROS production is recognized as necessary for all stages of the inflammatory process. Both innate and adaptive immunity entail redox-regulated processes, for instance, the governance of immune cells infiltration, their activation and differentiation, the oxidative burst of phagocytes, as well as the control of cellular signal transduction and transcription programs [3,4].

It is well established that the immune system plays a complex and dynamic role in cancer progression. In this regard, several studies have demonstrated its dual role due to host-protecting and tumor-sculpting actions [5,6,7]. Oxygen centered oxidants are formed by many cell types in the tumor microenvironment (TME), including cancer cells and innate and adaptive immune cells. ROS can be both beneficial and detrimental for the immune function, therefore they can indirectly impact cancer progression by shaping cancer immune surveillance. In this review, we describe the role of ROS in immunity and how they affect the antitumor immune response and discuss these effects in the context of disease progression and immunotherapy.

## 2. Reactive Oxygen Species Role in Immunity

ROS fulfill key functions in innate immunity as defense mechanisms and essential cell types involved in innate immune responses [8,9]. Several studies have found that ROS can function as direct chemoattractants, regulating immune cell recruitment. ROS promote immune cells infiltration in inflamed zebrafish tissue [10] or induce chemotactic proteins such as thioredoxin [11]. ROS not only control leukocyte recruitment but also their retention since myeloperoxidase (MPO)-derived ROS can promote paracrine neutrophil survival [12,13]. Multiple evidence showed that ROS are involved in sensing danger, that is, the presence of pathogens as well as tissue damage. Indeed, Pathogen-Associated Molecular Patterns (PAMPs) and Damage-Associated Molecular Patterns (DAMPs) recognition by immune cells can trigger intracellular signaling events leading to increased ROS generation that can result in inflammasome activation and pro-inflammatory cytokine production [3,4]. ROS play also a critical regulatory role in determining the initiation and outcome of phagocytosis. They are involved in the recognition and the engulfment of damaged cells [14] and phagocytic cells such as monocytes, macrophages and neutrophils produce ROS during the oxidative burst necessary for pathogens killing and damaged cells clearance [15]. Moreover, ROS are also involved in the signaling cascade leading to the formation of neutrophils extracellular traps (NETs), structures capable of entrapping and degrading microbes [16]. Notably, studies have shown that macrophage differentiation relies on ROS, although the process is not yet fully understood. Depending on the content of intracellular glutathione, the pro-inflammatory M1 and the anti-inflammatory M2 macrophages are characterized as oxidative and reductive macrophages, respectively, suggesting a redox regulation in their physiology [17,18]. ROS are also required for the function of another major effector of the innate immune system, Natural Killer (NK) cells—hydroxyl radical production is responsible for NK cells-mediated cytolysis, by promoting the secretion of cytotoxic factors from NK cells [19]. Dendritic cells represent a bridge between innate and adaptive immunity and play a key role in antigen-specific immune responses. ROS can trigger their differentiation from monocytes precursors or hematopoietic cells and induce their maturation by upregulating costimulatory molecules and enhancing their antigen-presenting capability [20]. Moreover, ROS regulate DC phagosomal pH and antigen cross-presentation [21]. 

Redox regulation of immune responses is not restricted to the activation and subsequent activity of innate immune cells. ROS are also instrumental for the activation of B and T cells, that is, for the generation of both humoral and cell-mediated adaptive immunity [22,23]. H_2_O_2_ plays an important role in B cell maturation, activation and B cell receptor (BCR) signaling [24,25]. Furthermore, antibodies secreted by plasma cells are capable of generating H_2_O_2_, which may help to kill antibody-coated cells [26]. It is well recognized that ROS levels increase after T cell activation by T cell receptor (TCR) signaling [27,28,29]. Acting as second messengers within T cells, they control cell proliferation and clonal expansion in response to infection or cancer [23]. Unsurprisingly, ROS play a pivotal role in the regulation of the differentiation and effector functions of various T cell subsets. For instance, a long-lived T helper (Th)2-skewed immune phenotype is favored by high microenvironmental levels of ROS [30]. By contrast, Th1 and Th17 phenotypes are promoted by low levels of ROS [31]. Cellular antioxidant mechanisms strictly control ROS levels to maintain effective T cell-mediated immunity. Indeed, ROS need to be compartmentalized during T cell activation, because deregulation of the mitochondrial pore permeability was shown to lead to increased cell death upon TCR stimulation [32]. Besides, prolonged ROS signaling can result in T cell hyporesponsiveness [33]. Lastly, ROS participate to T-cell balance under homeostatic and disease conditions by modulating T-cell apoptosis [34]. 

ROS represent pivotal mediators in the later stages of the immune response, which can involve not only the promotion of inflammation but also its resolution. Indeed, ROS influence and are released by regulatory T cells (Treg) and myeloid-derived suppressors-cells (MDSCs), which are important immune modulatory cells essential for the immune response control. Treg cell can suppress other T cells indirectly by their ability to prevent glutathione release from DCs [35] or directly by secretion of ROS [36]. Indeed, H_2_O_2_ was shown to inhibit Nuclear Factor κB (NF-κB)-induced cytokine expression from activated T cells [37,38]. The suppressive functions of MDSCs can be terminated by impeding their ROS production and MDSCs-derived ROS are reported to inhibit T cell response [39,40]. This phenomenon may result from the loss of TCR ζ chain expression caused by H_2_O_2_. MDSCs can also compromise TGF-β-induced Treg conversion from conventional T cells in a ROS-dependent fashion [41].

## 3. The Antitumor Immune Response and Cancer Immune Evasion Mechanisms: An Overview

It is well-established that host immune cells can both antagonize and stimulate cancer growth [42]. Indeed, their crucial involvement in tumor progression is acknowledged by the identification of inflammation and immune evasion as hallmarks of cancer [43]. Many inflammatory conditions can favor neoplastic transformation. However, whether or not inflammation is present in the origin of tumorigenesis, most tumors advance to a state of chronic inflammation that supports distinct aspects of cancer progression. Therefore, interactions between the immune system and the tumor take place in all different stages of the disease, from early events of neoplastic transformation to metastatic spreading and ultimately also during therapy [44].

During the early stages of tumor development, disease progression is controlled by the T-cell response against tumor-derived antigens, characterized by release of Th-1 cytokines, NK cells recruitment and the presence of CD8+ cytotoxic T cells (CTLs), which identify and kill the more immunogenic cancer cells (i.e., cancer immunosurveillance) [45]. Following the persistent selective pressure of the effector response, tumor subclones are selected and escape immune recognition and elimination by developing mechanisms that mimic peripheral tolerance [46]. At the same time, the tumor promotes the recruitment of CD4+ Tregs that neutralize anti-tumor immune cells. Moreover, as the tumor grows, it becomes hypoxic while the surrounding tissue becomes damaged, both of which are important signals for the recruitment of immune cells. Angiogenesis, extracellular matrix remodeling and immune evasion are influenced by tumor-associated macrophages (TAMs), tumor-associated neutrophils (TANs), myeloid-derived suppressor cells (MDSCs) and immature dendritic cells (DCs) and can accelerate tumor progression, metastasis and therapy resistance [47]. By contrast, the recruitment of cytotoxic macrophages and neutrophils, NK cells and mature DCs leads to the elimination of tumor cells in primary sites and after dissemination. Moreover, the immunogenic cell death in the inflamed tumor environment, which occurs in response to certain therapies, may result in antitumor adaptive immune responses [48,49].

Tumor cells evade the immune attack using two main strategies—eluding the immune recognition and prompting an immunosuppressive TME [50,51]. Malignant cells can express antigens that have the capacity to induce tumor-specific responses; however, the immune selection of cancer cells that lack or mutate immunogenic tumor antigens, as well as the acquisition of defects or deficiencies in antigen processing and presentation, may lead to loss of their antigenicity. Moreover, tumors can avoid elimination by diminishing their immunogenicity through the modulation of expression of costimulatory and coinhibitory molecules. Furthermore, some tumors evade immune elimination by disposing a suppressive microenvironment. 

## 4. Impact of ROS in Antitumor Immunity and Immune Escape

Considering what is known about ROS in tumorigenesis [52,53] and their influence in immunity, as described above, it is conceivable that both cancer immune surveillance and immune evasion display some degree of redox regulation ultimately shaping the cellular fate of tumor-infiltrating immune cells and cancer cells elimination. 

### 4.1. Impact of ROS on Tumor Antigenicity and Immunogenicity

**Figure 1:**
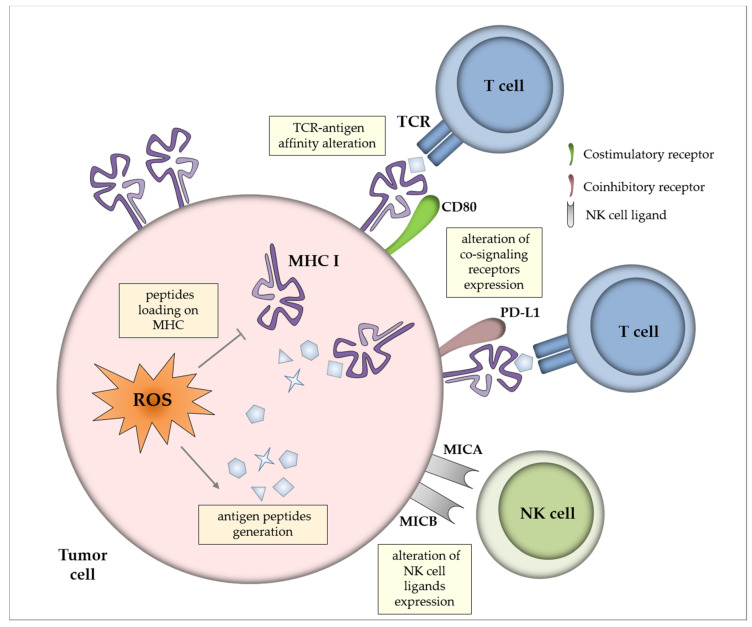
Impact of reactive oxygen species (ROS) on tumor antigenicity and immunogenicity. Oxidative stress in the tumor cells can induce the generation of antigenic peptides, which is counterbalanced by limited loading on major histocompatibility complex (MHC) molecules. Post-translational modification of proteins by ROS may alter antigenicity and modify T cell receptor (TCR) affinity to antigens. In addition, ROS may regulate the expression of co-signaling receptors for T cells (i.e., CD80 costimulatory molecule and PD-L1 coinhibitory molecule) and of NK cell ligands (i.e., MHC class I chain-related protein A and B MICA and MICB).

Tumor immunogenicity, which is the ability to induce adaptive immune responses, is dictated by two major criteria—antigen expression and antigen presentation (Figure 1). Weak antigenicity elicits a suboptimal immune response that provides the opportunity and time to tumor cells to develop immune evasion mechanisms [54]. Mapping of the subset of the immunopeptidome (the set of peptides selected and presented at the cell surface) that comprises redox-sensitive cysteine residues showed that a high proportion of cysteine-containing peptides are oxidatively modified physiologically [55]. In the context of tumor cells, alterations in the cellular redox state and the free oxygen radicals generated in inflammatory TME could yield post-translational modification of cysteine residues in proteins [56] which may alter antigenicity and have consequences for T cell escape. Indeed, the oxidative status of antigens can modify T cell receptor affinity to the antigenic peptide [55,57]. Moreover, oxidative stress triggers the upregulation of antigenic peptides generation that is compensated by a limitation of their capacity to be loaded onto major histocompatibility complex (MHC) molecules [58].

Alteration in the expression of co-signaling receptors for T cells is another strategy adopted by cancer cells to escape immune surveillance [59]. Lack of positive costimulatory ligands or the presence of inhibitory ligands on tumor cells have been suggested to participate to poor anti-tumor T-cell efficacy. Indeed, co-stimulation deficiency leads to anti-tumor T cells anergy, whereas in the presence of co-inhibitory signals T cells activation is suppressed [60]. ROS were shown to impact the expression of the coinhibitory molecule PD-L1 in cancer cells in vitro, although no simple and direct relationship could be deduced between elevation/reduction of ROS production and modulation of PD-L1 expression [61]. On the other hand, ROS could induce the expression of the costimulatory molecule CD80 via the c-Jun N-terminal kinase (JNK) and p38 mitogen-activated protein kinase (MAPK) pathways, that activated Signal transducer and activator of transcription 3 (STAT3) transcription factor in colon cancer epithelial cells in vitro [62]. Moreover, it appears that modest generation of ROS by cancer cells can trigger hypoxia [63], which can modulate immunity by regulating the expression of co-stimulatory (CD137, OX-40) and co-inhibitory (PD-L1) molecules for T and NK cell activation [64].

The presentation of antigens on MHC class I molecules is unnecessary for the identification of tumor cells by NK cells; thus tumor cells can still be eliminated even in the absence of proper antigen expression and presentation. Senescent myeloma cells upregulated ligands (MICA, MICB and PVR) for NK cell activating receptors Natural killer group 2 member D (NKG2D) and DNAX accessory molecule-1 (DNAM1) in an oxidant-dependent manner, resulting in enhanced NK cell activation [65]. Moreover, the upregulation of MICA and MICB gene expression was also shown in CaCo-2 colon carcinoma cell line upon oxidative stress [66], an effect that could strengthen NK cell recognition and tumor cell elimination.

### 4.2. Impact of ROS on Tumor Microenvironment

**Figure 2:**
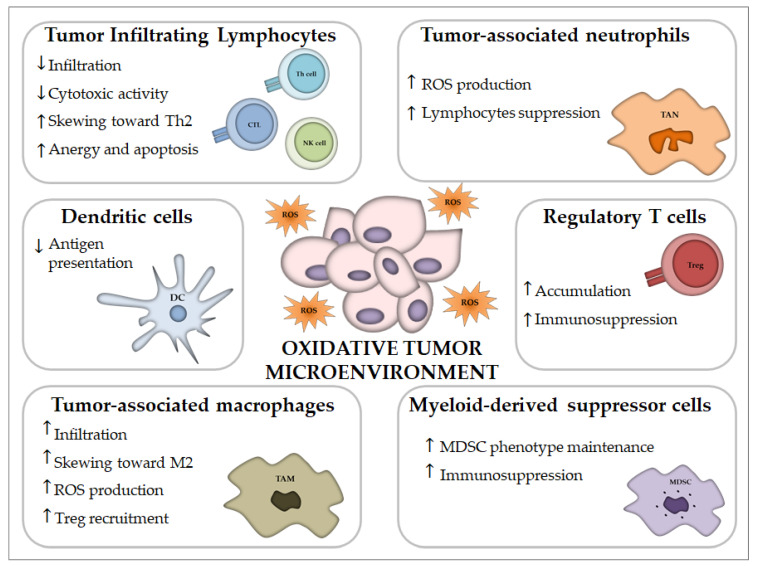
Impact of oxidative stress on immune cells of the tumor microenvironment. Oxidative stress in the tumor microenvironment promote immune suppression. It can reduce the infiltration of lymphocytes and favor the recruitment and accumulation of regulatory T cells and M2 tumor-associated macrophages. Antigen presentation by dendritic cells results impaired and tumor infiltrating lymphocytes are dysfunctional. Myeloid-derived suppressor cells and tumor-associated neutrophils inhibit lymphocytes functions through ROS production.

Cancer is associated with oxidative stress, mediated through ROS generated mainly by malignant cells, granulocytes, TAMs and MDSCs into the TME. The TME includes a large number of different immune cell types [67], among which MDSCs, TAMs and Tregs concurrently work to restrain the immune response to a tumor, allowing for greater tumor invasion, metastasis and resistance to treatments [68,69]. In this section, we will focus mainly on ROS functions and effects on the distinct tumor infiltrating immune cells which are essential to the host immune response to cancer (Table 1 and Figure 2).

#### 4.2.1. Tumor Infiltrating Lymphocytes (TILs)

TILs comprise cytotoxic lymphocytes, natural killer cells and T helper 1 lymphocytes which are pivotal for tumor cell recognition and elimination. As previously described, low levels of ROS are necessary for proper T cell activation, proliferation and differentiation, while high ROS have been noticed as one of the major factors for immunosuppression and inhibition for T cell activation and proliferation inside the TME [53,104]. TILs could be dysfunctional due to the ROS accumulated in the TME but they also demonstrated a persistent dysfunction of oxidative metabolism due to loss of mitochondrial function and mass when they infiltrated tumors, which led to impaired effector functions [88]. Moreover, T lymphocytes from peripheral blood of cancer patients showed an augmented ROS production compared to those of healthy subjects [105]. Cellular antioxidant levels resulted essential for maintaining the anti-tumor function of T cells within oxidative TME [34]. A study reported that central memory T cells characterized by higher cytosolic Glutathione (GSH), surface thiol and intracellular antioxidant levels could last for longer in an immunosuppressive microenvironment and better govern tumor growth than effector memory T cells, characterized by lower cytoplasmic antioxidant levels [89]. Indeed, a recent report showed that ROS scavengers could amplify the activation of CD8+ tumor-infiltrating lymphocyte in kidney tumors by activating the mitochondrial superoxide dismutase 2 (SOD2) [90]. Similarly, CTLs armed with engineered T cell receptors (CAR-T cells, chimeric antigen receptor-redirected T cells) that co-expressed catalase were secured from oxidative stress and preserved high tumor killing activity indicating that hydrogen peroxide participates to T cell anergy [106].

NK cells are innate lymphocytes able to constrain tumor development by their cytotoxic activity. However, tumor-infiltrating NK cells usually exhibit defective phenotypes and are characterized by either anergy or reduced cytotoxicity. Indeed, oxidative stress can alter natural killer cell functioning, contributing to immune escape within the TME. Hydrogen peroxide produced within TME inversely correlated with the infiltration of NK cells, possibly due to their preferentially induced cell death [83], whereas H₂O₂ derived from macrophages isolated from melanoma-bearing patients was demonstrated to reduce T and NK cells mediated cytotoxic activity [78]. Furthermore, tumor-produced ROS likely caused NK cell dysfunction in chronic myelogenous leukemia (CML), since catalase could restore NK cell cytotoxic capacity against primary tumor cells obtained from patients affected with this malignancy [79]. The inhibitory activity of ROS on NK cells recruitment was observed in melanoma and sarcoma mouse models [70], furthermore myeloid NADPH oxidase 2 (NOX2)-deficient mice diminished melanoma metastasis and increased Interferon gamma (IFN-γ) generation in NK cells, suggesting that myeloid-derived ROS hamper NK cells control of cancer malignancy [80]. Likewise, phagocytes derived ROS downregulated NKG2D and NKp46 surface expression in vitro, which has been suggested to mediate NK cell deficiency in patients with acute myeloid leukemia [81].

#### 4.2.2. Regulatory T Cells (Tregs)

Tregs are another immune cell type that is commonly present in the tumor TME. A rise in the number of Tregs in the TME denotes local immunosuppression, which is essential for cancer cells to escape from the immune system and represents an obstacle to cancer therapy [107]. Despite the deleterious effects of oxidative stress on natural killer (NK) and T cells, greater numbers of Tregs can be detected at tumor sites, indicating that Tregs can persist in this oxidant environment. Indeed, it was demonstrated that Treg cells, compared to effector CD4+T cells, are less sensitive to oxidative stress-induced cell death, a phenomenon that may be ascribed to their proven high antioxidative capacity [92]. However, it was recently discovered that tumor Treg cells sustain and amplify their suppressor capacity through death mediated by oxidative stress [93]. Furthermore, it was found that oxidative stress, rather than glycolysis, was the metabolic mechanism that controlled tumor Treg cell functional behavior and reinforced the therapeutic efficacy of immune checkpoint therapy [93].

#### 4.2.3. Myeloid-Derived Suppressor Cells (MDSCs)

MDSCs often represent the major producer of oxidizing species in the TME. In addition to their release of ROS, MDSCs often arise in oxidative-stress prone environments such as tumors. ROS not only initiate anti-oxidative pathways but also activate transcriptional programs that control the fate and function of MDSCs. Furthermore, MDSCs utilize redox mechanism to cause T cell unresponsiveness or T cell apoptosis and are reportedly more suppressive compared to granulocytes and monocytes from healthy subjects [94,95]. The maintenance of MDSCs in their undifferentiated state requires ROS molecules. Immature myeloid cells differentiated into macrophages when H_2_O_2_ was scavenged with catalase [102], while deficiency of NOX activity caused MDSCs to differentiate into macrophages and DCs in tumor-bearing mice [103]. Interestingly, lack of NOX2 activity in this model also impaired the ability of MDSCs to limit antigen-specific CD8+ T cell activation. Therefore, endogenous oxidative stress might represent a mechanism by which tumors inhibit the differentiation of MDSCs. 

MDSCs cause immunosuppression by T cell inhibition because ROS production inhibits recognition between TCR and MHC-peptide complex, as shown in a mouse lymphoma model [91]. In a mouse model, increased ROS levels in MDSCs suppressed IFNγ production and T-cell proliferation. Furthermore, MDSCs also inhibited T cells by exhaustion of cysteine and arginine (fundamental for T-cell activation and proliferation), generation of peroxynitrite (cytotoxic to T cells) and upregulation of the ROS-producing enzyme cyclooxygenase (COX)-2 in T cells [96,97,98,99]. More recently it was shown that tumor-induced MDSCs prevented T cell proliferation and promoted colorectal carcinoma cell growth through the production of ROS [100]. Interestingly, the use of ROS inhibitors completely abolished MDSCs immunosuppressive effects on T-cells [101]. Indeed, the co-culture of suppressed T cells and MDSCs from metastatic renal cell carcinoma, in the presence of the H_2_O_2_ scavenger catalase, could reinvigorate IFN-γ production in T cells to physiological levels [108].

#### 4.2.4. Tumor-Associated Macrophages (TAMs)

Macrophages are also among the first host cells infiltrating the tumor mass [109]. Their role in the TME is double-faced. On the one hand, macrophages have the potential to eliminate cancer cells. However, the appearance and the high number of macrophages in the tumor tissue is generally accepted as a negative prognostic marker. Depending on the composition of the microenvironment, macrophages may exist in many functional states. Generally, they are classified into two extremes—M1 and M2 macrophages [110]. M1 cells are classically activated cells that have a pro-inflammatory phenotype with antitumor activity, while M2 cells are alternatively activated cells that have immunosuppressive features promoting cancer progression. 

In lung and breast cancer models, ROS were essential for TAMs to invade the tumor niche and to acquire a pro-tumorigenic M2 phenotype [76]. Another study demonstrated that high intracellular ROS supported a more invasive phenotype in TAMs isolated from melanomas, possibly due to ROS-dependent tumor necrosis factor α secretion [77]. The authors of this study found that at least part of the intracellular oxidative stress was endogenously generated by TAMs from melanomas, which expressed elevated levels of several mitochondrial biogenesis and respiratory chain genes. Besides, macrophages-derived ROS drove the recruitment of Tregs to the TME for exerting tumor progressive roles [71]. Moreover, H_2_O_2_ production by macrophages has also been proven to sustain tumor progression in gastric cancer via modulation of miR-328-CD44 signaling [111].

#### 4.2.5. Tumor-Associated Neutrophils (TANs)

Tumor infiltrating neutrophils present functional heterogeneity and the existence of two polarized states, N1 and N2, was suggested similarly to macrophages [112,113]. N2-like TANs can show pro-tumorigenic activities whereas N1 exhibit cytotoxicity to tumor cells. Indeed, it was demonstrated that the infiltration of neutrophils in mouse tumor models induced tumor apoptosis with the use of ROS [72]. Furthermore, TANs could also impede metastatic dissemination in the lungs through hydrogen peroxide production [73]. Recently, ROS mediated cell elimination by TAN was shown to be dependent on tumor cell expression of TRPM2 [74]. Moreover, in mouse tumor models, TANs inhibited the proliferation of murine IL17+ γδ T cells via induction of oxidative stress, thereby preventing them from constituting the major source of pro-tumoral IL-17 in the TME [75]. On the other hand, ROS derived from neutrophil MPO could restrain NK cell activity against tumor cells [82] and could contribute to oxidative DNA damage and genetic instability [114]. Tumor cells can elicit c-Kit signaling in neutrophils, driving an oxidative phenotype that maintains ROS-mediated suppression of T cells even in the nutrient limited TME [115].

#### 4.2.6. Dendritic Cells (DCs)

DCs are crucial for eliciting anti-tumor immunity, due to their ability to (cross-)present antigens and activate T cells. This capacity is affected by the inflammatory environments that the cells meet [116]. The effects of ROS on DCs are complex, including metabolic and transcriptional changes that can affect the quality of DCs [84,117]. Although DCs actively utilize endo/phagosomal ROS to assist cross-presentation, the augmentation of the environmental redox potential could also hamper cross-presentation [85]. Excessive ROS can lead to chronic ER stress responses and oxidative damage to intracellular lipids that can inhibit DCs capacity to present local antigens to intratumoral T cells [86,87], thereby impairing the development of an effective antitumor immune response.

## 5. Impact of ROS on Cancer Immunotherapy

Much evidence points out that an oxidative milieu has an enormous impact on tumor cells, as well as TILs and other immune cells (and their interactions). Thus, it is plausible that ROS may also have a role in the efficacy of novel cancer immunotherapy approaches, not only in conventional anticancer treatments [2,118,119].

Immune checkpoint inhibitors (ICI) and adoptive cell therapy (ACT) are two of the main actors in the immune-oncologic approach aiming at boosting antitumor immunity [120]. Treatment strategies based on antioxidants exploitation were developed to maintain antitumor activity by ACT under hypoxia and oxidative stress conditions. Indeed, exposure of ex vivo expanded TILs to N-acetyl cysteine treatment avoided their apoptosis following adoptive transfer into patients, eventually supporting extended survival of patients receiving them [121]. Moreover, Ligtenberg et al. remolded CAR-T cells to co-express CAT for improving their antioxidant capacity [106]. These CAR-CAT-T cells had a reduced oxidative state at both the basal state and upon activation but they preserved their antitumor activity. Moreover, they could exert bystander protection of T cells and NK cells even in the presence of high H_2_O_2_ concentrations. Another strategy of cancer immunotherapy is the repolarization of immunosuppressive TAMs to antitumor M1 macrophages [122]. TAM-targeted ROS-inducing micelles effectively repolarized TAMs to M1 macrophages and largely augmented the activated NK cells and T lymphocytes in B16-F10 melanoma tumors, causing vigorous tumor regression [123].

In the last decade, ICI targeting Programmed cell death protein 1 (PD-1)/PD-L1 blockade significantly increased the survival rate in cancer patients, revolutionizing the landscape of cancer treatment. Recently, it was reported a synergetic effect of mitochondrial activation chemicals with anti PD-1 therapy on induction of T cell-dependent antitumor activity [124]. The authors showed that tumor-reactive CTLs, isolated from mice treated with anti PD-L1, carried higher levels of ROS and ROS generation enhanced the tumor killing activity of PD-1 blockade by the expansion of effector/memory cytotoxic CD8+ lymphocytes. Thus, altering endogenous mitochondrial activity in CTLs may affect the response to PD-1 blockade. Another study showed a correlation between the ability of mouse tumor cell lines to consume oxygen and produce hypoxic environments with their sensitivity to PD-1 checkpoint blockade [125], thus suggesting that decreased levels of ROS and consequently a less hypoxic TME may intensify the effectiveness of PD-1 blockade immunotherapy. Finally, a recent study reported that continuous NOX4-dependent ROS generation was required in cancer-associated fibroblasts (CAF) to maintain their activated phenotype, which promoted resistance to different immunotherapy modalities. Specifically targeting CAF NOX4 could re-sensitize CAF-rich tumors to anti-cancer vaccination and anti PD-1 checkpoint inhibition by reshaping CAF-regulated immune microenvironment [126].

## 6. Conclusions

Taken together, the data presented in this review uncover a double-faced role of ROS in the antitumor immune response. Additional studies are needed to characterize how different subcellular localization, magnitude and duration of ROS production within tumor infiltrating immune cells and into the TME are affecting tumor immunity. Cancer treatment approaches via oxidative or antioxidative drugs should consider the broad range of both beneficial and detrimental effects of ROS on immunity and cancer progression. A more successful strategy could be to target ROS or antioxidants to a specific cell type and conceive innovative combinatorial therapies. Moreover, further studies addressing the potential role of ROS levels and redox status as prognostic or predictive markers of immunotherapy outcome are warranted.

## Figures and Tables

**Table 1:** Summary of ROS Functions and Effects on Immune Cells.

Cell	Effects of Reactive Oxygen Species	References
Leukocytes	Recruitment to inflamed tissues	[10,11,12,13,70,71]
Phagocytes	Recognition and engulfment of damaged cells	[14]
	Pathogen killing and damaged cells clearance	[15]
Neutrophils	Neutrophil Extracellular Traps (NET) formation	[16]
	Cytotoxic activity	[72,73,74,75]
Macrophages	Inflammasome activation	[3,4]
	Polarization	[17,18,76,77]
	Tregs recruitment	[71]
NK cells	NK cells-mediated cytolysis	[19]
	Suppression of NK functions	[70,78,79,80,81,82]
	Induction of apoptosis	[79,83]
DCs	Differentiation from precursors	[20]
	Maturation	[84]
	Antigen cross-presentation	[21,85,86,87]
T cells	Activation	[22,23,27,28,29]
	Differentiation	[30,31]
	Inhibition	[33,37,38,78,88,89,90,91]
	Apoptosis	[32,34]
B cells	Activation and differentiation	[24,25]
	Pathogen elimination by antibodies production	[26]
Tregs	Accumulation in oxidative microenvironment	[92]
	T-reg mediated suppression	[35,36,93]
MDSCs	MDSC-mediated immunosuppression	[39,40,41,94,95,96,97,98,99,100,101]
	Maintenance of MDSCs phenotype	[102,103]
